# Short-term evaluation of motor and sensory nerve conduction parameters in COVID-19-associated peripheral neuropathy patients

**DOI:** 10.1186/s43168-023-00199-1

**Published:** 2023-05-10

**Authors:** Mahmood D. Al-Mendalawi

**Affiliations:** Department of Paediatrics, Al-Kindy College of Medicine, University of Baghdad, Baghdad Post Office, P.O.Box 55302, Baghdad, Iraq

To the Editor,

On using motor and nerve conduction studies, Shaddad et al. [1] found that motor nerve conduction (MNC) latency and amplitude for the median nerve (MN) were deranged significantly in COVID-19 patients. The COVID-19 patients also had significantly greater MNC latency and F wave latency for the posterior tibial nerve (PTN). The D-dimer and C-reactive protein levels were correlated with a significant positive association with a latency of MN MNC, sensory nerve conduction (SNC), and f-wave; latency of MNC and F wave of the PTN; and SNC latency for sural nerve [1]. In addition to the study limitation of the lack of long-term follow-up which was addressed by Shaddad et al. [1], we, hereby, explore the following limitation. Neurophysiological studies, which involve various components, are crucial tools for the investigation of neuromuscular diseases. They could provide useful information on peripheral nerve function and knowledge of the reference values (RV) of these studies is critical for clinicians in evaluating suspected neuromuscular disorders. As these RV are impacted by numerous biological (such as age, gender, body height), physical (such as the temperature of the limb), and technical factors [2, 3], different populations-specific RV of motor and nerve conduction studies have been formulated to be utilized in health centers and research [4, 5]. Interestingly, Egypt has developed its own RV for these studies [6]. In the study methodology, Shaddad et al. [1] stated that the values below the 95^th^ centile or ± 2SD of control for all components of neurophysiological studies were regarded as abnormal. However, they didn’t address the reference of the employed RV. Due to this limitation, we believe that the study findings presented by Shaddad et al. [1] are doubtful.

## Data Availability

Not applicable.

## Abbreviations

MNC: Motor nerve conduction
MN: Median nerve
PTN: Posterior tibial nerve
SNC: Sensory nerve conduction
RV: Reference values

## References

[1] Shaddad AM, Hussein AARM, Tohamy AMA, Khalil WGE (2023). Short-term evaluation of motor and sensory nerve conduction parameters in COVID-19-associated peripheral neuropathy patients. Egypt J Bronchol.

[2] Kiss G (2005). Basic neurography and its diagnostic importance. Ideggyogy Sz.

[3] Tavee J (2019). Nerve conduction studies: basic concepts. Handb Clin Neurol.

[4] Shivji Z, Jabeen A, Awan S, Khan S (2019). Developing normative reference values for nerve conduction studies of commonly tested nerves among a sample pakistani population. J Neurosci Rural Pract.

[5] Kim JY, Kim E, Shim HS, Lee JH, Lee GJ, Kim K, Lim JY, Beom J, Lee SY, Lee SU, Chung SG, Oh BM (2022). Reference standards for nerve conduction studies of individual nerves of lower extremity with expanded uncertainty in healthy Korean adults. Ann Rehabil Med.

[6] Khafagi AT, Hamdy NA, Hasan EM, Ismail MM, Abdelkader MM, Saleh RN, Mohamed RB (2016). Standard norms of nerve conduction studies in a sample of healthy individuals in Minia Governorate. MJMR.

